# Breastfeeding in Phenylketonuria: Changing Modalities, Changing Perspectives

**DOI:** 10.3390/nu14194138

**Published:** 2022-10-05

**Authors:** Juri Zuvadelli, Sabrina Paci, Elisabetta Salvatici, Federica Giorgetti, Graziella Cefalo, Alice Re Dionigi, Valentina Rovelli, Giuseppe Banderali

**Affiliations:** Clinical Department of Pediatrics, San Paolo Hospital, ASST Santi Paolo E Carlo, University of Milan, Via Antonio di Rudinì 8, 20142 Milan, Italy

**Keywords:** phenylketonuria, PKU, dietetics, childhood nutrition, breastfeeding

## Abstract

Phenylketonuria (PKU) management aims to control phenylalanine (Phe) intakes. In newborns and infants this implies possible titration of Human milk (HM) with supplementation of Phe-free formula. HM benefits, better if prolonged, are well known in healthy populations, suggesting it may be used in PKU patients. Despite that, the current literature does not define recommendations on how best perform it in such a population. The main purpose of this study was to evaluate nutrition approaches in newborns and infants affected by PKU and to define if differences can influence the duration of breastfeeding. Data from 42 PKU infants were reviewed. Of these, 67% were breastfed with the use of three different approaches. The type of approach used impacted the duration of breastfeeding, which was longer when using a pre-measured amount of Phe-free formula administered prior to HM. This is the first study to suggest a specific method for breastfeeding in PKU. Considering widely known breastfeeding benefits, both for patients and their mothers, our data should encourage adequate awareness on how to choose correct breastfeeding modalities.

## 1. Introduction

In phenylketonuria (PKU) there is a toxic phenylalanine (Phe) accumulation that damages mostly the central nervous system, resulting in progressive and irreversible cognitive impairment. Diagnosis is made soon after birth thanks to newborn screening tests with treatment beginning immediately thereafter, when required, consisting of a lifelong dietary intervention providing low Phe intakes; frequent blood Phe values monitoring allows for confirmation of the adequacy of the treatment [1,2,3,4,5,6,7].

Breastfeeding is a well-known ideal food to guarantee optimal growth and development of healthy infants and children with many demonstrated advantages possibly related to the content of long-chain polyunsaturated fatty acids and immunoglobulins, highly bioavailable iron and non-protein nitrogen combinations, such as lactoferrin polyamines and nucleotides; it also seems to ameliorate emotional attachment and satisfaction [8]. In addition to the known nutritional components of human breast milk, i.e., water, carbohydrates, fats and proteins, it is also a rich source of microRNAs, which impact epigenetic mechanisms [9] and immune-related health outcomes later in life, including immunoglobulins, cytokines, chemokines and growth factors [10,11].

With regards to PKU, interest in human milk (HM) Phe content started during late 1970s, when researchers succeeded in determining that it has lower concentrations than standard formula (2482 μmol/L vs. 4419 μmol/L) and that it can allow Phe levels more steady in range compared to formula administration [2]. It was then concluded that HM can be used in the dietary intervention of infants with PKU alongside Phe-free formula [2,12,13,14,15,16], but, to date, no specific recommendations on how to correctly use it in such a population, nor how to handle it with the associated use of Phe-free medical formulas, have been reported, nor have papers tried to compare different modalities of approach and how they may affect breastfeeding duration, blood Phe control and growth.

Mixed feeding is an option that can be used. Regarding this perspective, the used and reported techniques include: (1) administering a pre-measured amount of pumped HM prior to Phe-free formula (Bottle-fed); (2) administering a pre-measured amount of Phe-free formula prior to HM to be administered until satiety (PS pre-fed); (3) administering a pre-measured amount of Phe-free formula prior to “time-controlled” breastfeeding (meaning a fixed meal duration time); (4) administering alternate feeds of HM and Phe-free formula throughout the day (Alterne-fed) titrating milk amounts and managing meal frequencies. Among these, the most common practice to date seems to administer a pre-measured amount of Phe-free formula prior to free breastfeeding [17,18,19,20,21,22,23].

As a matter of fact, approaches to breastfeeding and Phe-free medical formula use may vary throughout clinical settings, countries and also physicians dealing with PKU patients, and the lack of specific indications for the practical care of newborns during the feeding process may result in different outcomes both from a nutritional and developmental standpoint, including possible correlations with the type of method selected. 

To date, breastfeeding in the PKU population still appears as an open challenge. Alongside its common possible related issues, as described in the general population (such as supply issues, eventual need of breast pumping, nipple confusion, poor latch, breast pain, mastitis, etc.) [24], in PKU patients, the strong stress and anxiety component related to mother’s newly acquired awareness of the disease, turning a natural and physiological act into a treatment delivery, must be considered. Furthermore, the use of a breast pump may be strictly required in such a context, leading to a loss of physical contact with the newborn or, if more empirical methods are used, such as a pre-measured amount of Phe-free formula administered prior to breastfeeding until satiety, it is possible for mothers to experience fear of not being capable to provide adequate amounts of HM [16,17,25,26,27].

This is the first study to attempt to evaluate and compare different breastfeeding practices in newborns with PKU, with the main purpose of clarifying if different approaches can influence HM availability and possibly result in long-term changes of growth parameters and/or indices of metabolic control.

## 2. Patients and Methods

### 2.1. Study Design and Data Collection

Starting from 2012 to 2021, data from medical records of patients admitted to the Clinical Department of Pediatrics, San Paolo Hospital, ASST Santi Paolo e Carlo, University of Milan, Italy, for newborn screening results demonstrating blood Phe values ≥360 μmol/L, therefore suspected for PKU and requiring dietary intervention (diagnosis was confirmed genetically), were retrospectively reviewed and analyzed by means (age range 0–12 months). The objects of collection were multiple data, including breastfeeding modalities, anthropometric measures, phe values, dietary regimens, type of delivery, nationality and parity. Blood Phe values were obtained from DBS analysis using tandem mass spectrometry (MS/MS).

With regards to breastfeeding modalities, two groups were identified, a “HM-Fed group” (receiving HM) and a “F-fed group” (receiving formula milk, at least when starting PKU dietary intervention). As suggested by literature, the “HM-Fed group” was also divided into subgroups according to the method used for breastfeeding, as follows: (1) administering a pre-measured amount of pumped HM prior to a Phe-free formula (Bottle-fed); (2) administering a pre-measured amount of Phe-free formula prior to HM to be administered until satiety (PS pre-fed); (3) administering a pre-measured amount of Phe-free formula prior to “time-controlled” breastfeeding (meaning a fixed meal duration time); (4) administering alternate feeds of HM and Phe-free formula throughout the day (Alterne-fed) titrating milk amounts and managing meal frequencies.

The choice of the breastfeeding strategy to be applied was made by the dietitian from the multidisciplinary team in accordance with the metabolic indication and mother’s preference, taking in account pre-treatment Phe levels for safety purposes. Observation of the breastfeeding modality lasted until 12 months of age.

With regards to anthropometric measurements, indices of weight and length were collected at recurring intervals, matched with the previewed evaluations at the clinic (birth, 3, 6, 9 and 12 months of life). For better interpretation, WHO (World Health Organization) z-score growth curves were calculated.

With regards to Phe values, pre-treatment Phe values were used to classify different phenotypes (>1200 μmol/L, classic PKU; 600–1200 μmol/L, mild PKU; 360–600 μmol/L, HPA) [28]) and data were reviewed thereafter including results from dried blood spot (DBS) and plasma amino acids [1].

With regards to dietary regimens, these were reviewed for the entire duration of the study period and all delivered by the same metabolic dietician. Data for energy (Kcal/Kg/day), proteins (g/kg/day) and Phe intakes (mg/kg/day) were collected. If applied, data from the “wash-out period” (a dietary approach characterized by an intake of 0 mg of Phe/day lasting 24 to 72 h in case of pre-treatment Phe values > 1000 μmol/L [29]) were also reviewed and analyzed.

The dietary Phe intakes were obtained from what was included in the provided diet, also confirmed by data derived from food dietary records routinely filled in by families during clinical evaluations. An average Phe content of 46 mg/100 mL for HM has, in fact, been described in the current literature, thus this was considered as the reference in accordance with what is indicated in the European guidelines [1]; for formula milks, data provided by the companies within labels were used as references.

Mothers were also screened for possible maternal PKU with a plasma amino acid assay at time of hospitalization, as suggested by national guidelines.

Patients with incomplete records or insufficient data, affected by other known chronic conditions, with birth weight <2500 g or gestational age <37 weeks were excluded from this study, with the aim to collected homogeneous subjects and avoid weight-related bias. 

### 2.2. Statistical Analysis

Statistical data analyses were performed using R version 4.0.3 (Team R Development Core, 2018) implemented with “rcmdr”, an open-source software and flexible programming language used for the statistical data analysis as well as graphic creations. All data are reported as means ± SD or numbers (%) unless otherwise indicated. The differences between studied groups were determined by Mann–Whitney U test or Kruskal–Wallis test. Spearman’s rank correction coefficient was used to measure the statistical dependence between two variables. Repeated-measures ANOVA test was performed to evaluate the effect of different types of interventions in the groups over time. Significant values were considered for *p* < 0.05.

## 3. Results

Forty-two PKU subjects (57.1% male, *n* = 24) were identified at newborn screening for blood Phe values ≥ 360 μmol/L (mean 1236 ± 760 μmol/L) during the study period. Of these, 78.6% of patients (*n* = 33) were born from vaginal delivery, while the remaining part were born by C-section. Regarding the mothers, 64.2% were Italian (*n* = 27) and 35.7% were primiparous (*n* = 15). None of the mothers had PKU.

All patients included in this study required dietary intervention, which consisted of three different Phe-free formulas used with comparable nutritional characteristics. Based on pre-treatment Phe values, clinical phenotype results were distributed as follows: classic PKU 42.9% (*n* = 18), mild PKU 35.7% (*n* = 15) and HPA 21.4% (*n* = 9). Of the patients, 45% (*n* = 19) required a period of “wash-out” at time of admission, lasting 55.5 (±16.9) hours on average. 

Diet start occurred at a mean age of 12.7 ± 3.4 days (mean 16.6 ± 2.4 days in the first five-year study period, 2012–2016, while it was 9.7 ± 3.6 days in the second five-year period, 2017–2021, following adaptations to European Guidelines [1]). On average, 2.3 ± 0.9 days resulted elapsing from the telephone call communicating the positivity of the newborn screening to the time of admission to the hospital. During this time lapse, HM rates seemed to decrease: upon discharge from birthplace, 88% of PKU (*n* = 37) were breastfed, while upon admission to our department only 67% (*n* = 28) were still on HM, even if there were no reports of possible health problems that could have hindered breastfeeding (e.g., nipple ulcers, obstruction of the milk ducts, mastitis).

Dietary interventions were prescribed according to the patient’s phenotype, thus regulated to achieve blood Phe concentrations of 120–360 μmol/L, as suggested by European guidelines [1]. This resulted in a mean prescribed amount of 45.7 ± 13.0 mg Phe/Kg/day during the first month of life, decreasing by up to 39.1 ± 12.6 mg Phe/Kg/day at the end of study period (12 months of age). Average dietary protein intake during the study period resulted 2.3 g/Kg/day (±0.2) without relevant changes from diet start (2.5 g/Kg/day ± 0.5) to end of study period (2.3 g/Kg/day ± 0.2). Average energy intake measured at the beginning of the diet was 120 kcal/kg/day (±11), while it was 100 kcal/kg/day (±12) at end of study (12 months) with a gradual and linear decrease over months.

**Figure 1:**
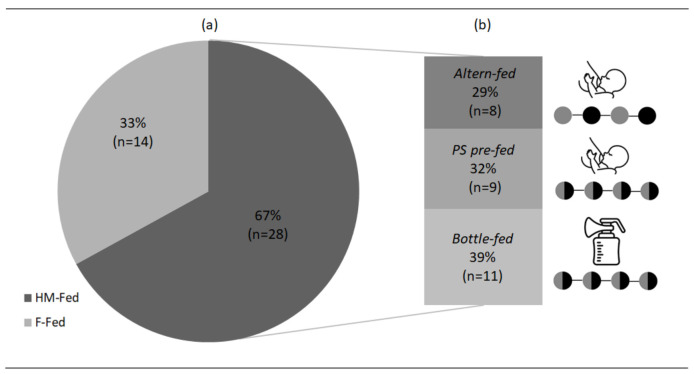
(**a**) Representation of patients fed with HM and patients fed with formula, (**b**) subgroup of patients fed with HM subdivided with the different methods.

Most part of patients included in this study were breastfed at diet initiation (HM-Fed group, *n* = 28, 67% vs. F-Fed group, *n* = 14, 33%). Among the mostly used breastfeeding strategies in the literature, only three were applied among our study population and included bottle-fed (39%, *n* = 11), PS pre-fed (32%, *n* = 9) and Alterne-fed (29%, *n* = 8); the chosen method to breastfeed was established at the beginning of the treatment and remained unchanged during the first year of life. Data are summarized graphically in Figure 1.

**Figure 2:**
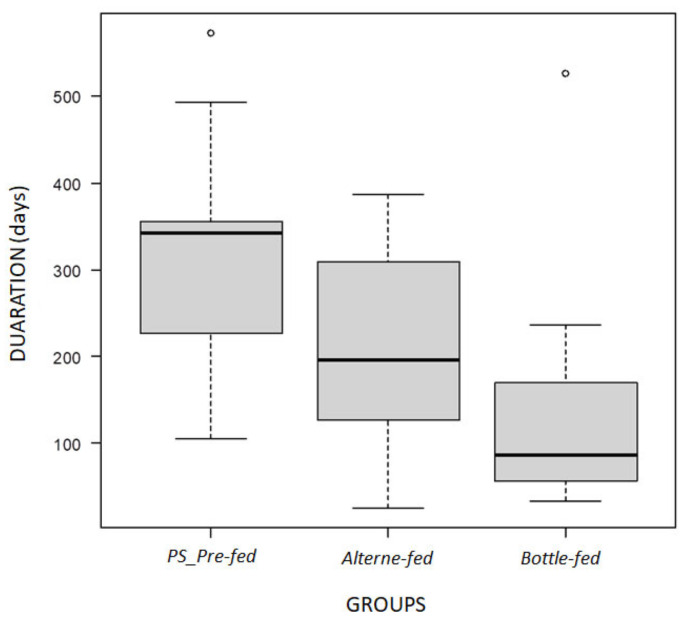
Breastfeeding duration in PKU patients who received HM with different strategies.

Breastfeeding rates decreased over time: 50% of patients were still breastfed at the age of 3 months, while only 33.3% were breastfed at 6 months, 21.4% at 9 months and 14.3% at 12 months of life. Average breastfeeding length was 7.2 months (range 0.8–18.8) and this did not change based on possible considered variables, such as mother’s ethnicity, type of delivery or pre-treatment Phe values. Besides that, a significant difference was instead found according to the used method for breastfeeding. In particular, a longer duration of breastfeeding was detected in the PS pre-fed group compared to both Alterne-fed and Bottle-fed (mean 313.0 ± 157.04 vs. 209.0 ± 127.1 vs. 146.3 ± 141.6 days, respectively), as represented in Figure 2. This difference was statistically significant (*p* = 0.04).

With regard to indices of metabolic control, these results were adequate throughout the study period, with mean Phe values = 198,1 ± 46.1 μmol/L calculated on a total of *n* = 2995 DBS retrospectively reviewed. In particular, 90.1% of analyzed DBS resulted <360 μmol/L and every subject, also taken alone, did show mean Phe values <360 μmol/L. 

The use of HM vs. formula did not change the metabolic outcomes (*p* = 0.594); likewise, the use of the three different methods of breastfeeding (Bottle-fed, PS pre-fed, Alterne-fed) did not show significant differences in terms of related Phe values (*p* = 0.141) and, even if the pre-treatment Phe value differed between subgroups (respectively, 1558–819–1235 μmol/L), the difference between means did not reach the statistical significance (*p* = 0.06). 

**Figure 3:**
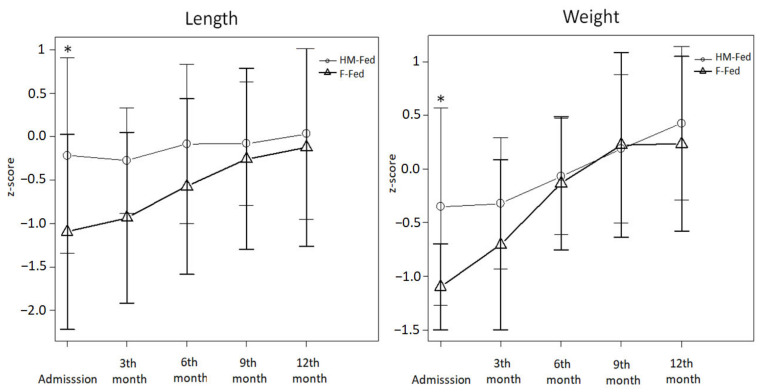
Length and Weight growth curve (WHO) of breastfed (HM-fed) and non-breastfed (F-Fed) PKU infants. Repeated-ANOVA measures were performed to study the variables at each time with data grouped into two categories. For the length variable no statistically significant association can be found between groups and time (*p*-value 0.197), for the weight variable statistically significant association can be found between groups and time (*p*-value 0.004). We also performed the Mann–Whitney U test (* in figure) each time, finding a significant difference for both length and weight upon admission to our clinic (respectively, *p* = 0.03 and *p* = 0.004).

Lastly, with regards to anthropometric measurements, the mean birth weight was 3.033 ± 436 g among our patients, thus it was normal, on average, compared to general population. Deepening the results, it could be highlighted that the F-fed group demonstrated lower weight values at birth than the HM-fed group (2918 vs. 3106 g; *p* = 0.04). Besides that, weight and length z-scores have shown recovery trends following clinical evaluations (from −0.62 to 0.30 and from −0.5 to −0.1 at admission and at 12 months of life, respectively). Analysis of growth parameters divided per different population, according to the type of milk received are described in Figure 3.

As represented, our sample had a z-score (both for weight and length) that shifted on the lower percentiles in both groups, with a significant difference between HM-Fed and F-fed groups upon admission to our pediatric unit. On the other hand, groups, overtime, moved in the direction of the median for both parameters. 

Length’s speed of growth was not significantly influenced by the type of milk used, however, there was a significantly greater growth in children who received Formula-milk (*p* = 0.004) with regards to weight.

Growth was also assessed in the sub-groups (Bottle-fed, PS pre-fed, Alterne-fed): significant associations between groups and time could not be found. 

## 4. Discussion

Dietary management practices in PKU patients during the first year of life are currently extremely heterogeneous. At the same time, little data are available [17,18] on how possible changes in the approach can lead to different effects. In this regard, we mean both the lack of data relating to the type of food administered and its outcomes (breast milk or infant formula, based on phenotype), and also on the different possible methods of administration that could lead to possible differences, even in the long term. This retrospective study is the first to try to elucidate such aspects, also comparing groups of patients who were breastfed differently during the first year of life.

As preliminary results, we can state that breastfeeding rates upon discharge from birthplace in our sample of PKU patients were slightly lower than the national average. In particular, 12% of PKU patients were not breastfed upon discharge from birthplace (vs. 9% on national average [30]) and this prevalence increased up to 33% upon admission to the clinic, after the communication of positive newborn screening has been carried out. These data are not reassuring and prompt us to think of this time lapse as a relevant window of action when dealing with nursing mothers of PKU patients. This is also based on the fact that no other possible health problems that could have hindered breastfeeding (e.g., nipple ulcers, obstruction of the milk ducts, mastitis) were reported, thus this significant reduction may be, at least partially, affected by the stress that families suffer after they are called back in view of a positive screening, and by mother’s fear of harming their child through breastfeeding. These data show the need to support these families, not only after the diagnosis, but also in that short period of time between the notification of a positive result and the eventual hospitalization [31].

Breastfeeding duration was measured in all patients, resulting in an average of 7.2 months length, which is slightly lower than 8.3 months in the general population [32]. Assessing breastfeeding rates over time, we observed that about 33% of our patients were still fed with HM at the age of 6 months. These data seem to be in line with the results of a recent survey carried out at the European level, which precisely shows results around 30% [33], and demonstrates a considerable increase when compared to a previous study carried out at the end of the 1990s in PKU patients in follow up at our clinic [34]. In that case, the prevalence of HM was only 9% at 6 months of age and this may lead to the consideration that a lot of efforts have been put into supporting mothers during recent years, as demonstrated by the fact that, in our sample, HM was maintained in all available cases and mothers were encouraged to continue overtime despite PKU phenotype, also pumping milk as needed. This has obviously led to an improvement in the overall data, even though there is still space for trying to reach rates found in the general population (50 vs. 68.3%, 33.3 vs. 60.9% and 14.3 vs. 32.2% at 3, 6 and 12 months of age, respectively [35]).

Aiming to assess whether any specific variables could influence breastfeeding duration, based on our reported results, we can also define that breastfeeding modality can, in fact, have an important impact on breastfeeding duration. Generally speaking, duration was longer when applying methods that involve suckling at the breast, and it was more evident when patients were PS pre-fed compared to both Alterne-fed and Bottle-fed (mean 313.0 ± 157.04 vs. 209.0 ± 127.1 vs. 146.3 ± 141.6 days, respectively), as represented in Figure 2; this difference was statistically significant (*p* = 0.04). Such data are particularly interesting, also considering that Bottle-fed children’s mothers are educated to store the milk produced in excess at home at temperatures of −18 °C for up to 6 months, continuing to feed the baby with HM even after the milk production has stopped. We believe that these data are very relevant, since it is the first time that this can be described, and also because they did not reveal any disadvantage with regards to metabolic control (which was adequate throughout the entire study period with no changes in the metabolic outcome based on type of milk nor modality of its administration), despite a significant advantage to breastfeeding duration. These results should push metabolic centers to reflect on the possibility of questioning the dietary management of children in the first months of life, assuring HM if available and being in favor of a PS pre-fed modality of administration.

With regards to nutritional assessments, in our sample, prescribed amounts of Phe, along with adequate intake of calories in line with current guidelines, allowed satisfactory metabolic control and growth over time. Total protein amounts prescribed were slightly higher than those recommended by the European guidelines, even if it is common practice for all metabolic clinics to maintain a protein intake >2 g/kg/day [1,36]. 

As for growth, we observed that our population was, on average, in the lower percentiles. The weight at admission of the F-fed group was significantly lower than the HM-fed group: 35% of F-fed children were formula-fed from the first days of life, while the remaining part had been discharged from the birth unit with breastfeeding and switched to Formula-milk due to low maternal lactation. This event may have had a temporary impact on the growth, as demonstrated by the fact that at other time points the groups tend to normalize. Specifically, the F-Fed group seems to grow more in weight than HM-fed and this could be linked to the use of formula milk which, like in the general population, could have increased the growth rate in the first months; also, it could be related to a physiological mechanism of “catch-up growth” [37,38].

As for the growth in length, this appears to follow the same pattern, although it does not appear to be significantly different depending on the type of milk.

One limit of this study is that we were unable to assess the satisfaction and anxiety of mothers during breastfeeding, an aspect that we intend to investigate moving forward. We will also expand the clinical records and involve other centers of the national territory. 

## 5. Conclusions

The promotion of breastfeeding due to its known positive effects on the healthy population is a well-known topic. Nevertheless, expert in metabolism clinicians’ positions on promoting it even in patients with phenylketonuria are not clear, despite possible advantages. There are also no known recommendations on how to manage it in the safest and most effective way possible, in order to obtain a longer duration and a better result on differential parameters.

To the best of our knowledge, this is the first paper that attempts to compare the main methods of breastfeeding in the PKU population and evaluate possible consequences on its duration, rates and possible outcomes.

Our results encourage clinicians to promote HM use even in PKU patients, in order to obtain its most positive effects, as already widely described in the general population; furthermore, they should promote administration of HM it using the PS pre-fed method, which is likely also the most physiological method capable of a more uniform distribution of natural proteins over 24 h, and allows the baby obtain close touch and bonding with its mother. 

Further studies will be needed to further confirm our evidence and possibly expand it for possible further applications. However, we believe that these results represent the first fundamental step in providing PKU infants the best start in life.

## Data Availability

Data are available on request due to privacy restrictions. The data presented in this study are available on request from the corresponding author. The data are not publicly available due to internal regulations.
